# Investigating female versus male differences in white matter neuroplasticity associated with complex visuo-motor learning

**DOI:** 10.1038/s41598-024-56453-z

**Published:** 2024-03-11

**Authors:** Eric D. Kirby, Justin W. Andrushko, Shie Rinat, Ryan C. N. D’Arcy, Lara A. Boyd

**Affiliations:** 1 BrainNet, Health and Technology District, Vancouver, BC Canada; 2 Faculty of Individualized Interdisciplinary Studies, Simon Fraser University, Burnaby, BC Canada; 3 Faculty of Science, Simon Fraser University, Burnaby, BC Canada; 4 DM Centre for Brain Health, Faculty of Medicine, University of British Columbia, Vancouver, Canada; 5 Department of Sport, Exercise and Rehabilitation, Faculty of Health and Life Sciences, Northumbria University, Newcastle Upon Tyne, UK; 6 Brain Behaviour Laboratory, Department of Physical Therapy, Faculty of Medicine, University of British Columbia, Vancouver, Canada; 7 Faculty of Applied Sciences, Simon Fraser University, Burnaby, BC Canada

**Keywords:** Myelin biology and repair, Learning and memory, Motor control, Imaging techniques, Neurology

## Abstract

Magnetic resonance imaging (MRI) has increasingly been used to characterize structure–function relationships during white matter neuroplasticity. Biological sex differences may be an important factor that affects patterns of neuroplasticity, and therefore impacts learning and rehabilitation. The current study examined a participant cohort before and after visuo-motor training to characterize sex differences in microstructural measures. The participants (N = 27) completed a 10-session (4 week) complex visuo-motor training task with their non-dominant hand. All participants significantly improved movement speed and their movement speed variability over the training period. White matter neuroplasticity in females and males was examined using fractional anisotropy (FA) and myelin water fraction (MWF) along the cortico-spinal tract (CST) and the corpus callosum (CC). FA values showed significant differences in the middle portion of the CST tract (nodes 38–51) across the training period. MWF showed a similar cluster in the inferior portion of the tract (nodes 18–29) but did not reach significance. Additionally, at baseline, males showed significantly higher levels of MWF measures in the middle body of the CC. Combining data from females and males would have resulted in reduced sensitivity, making it harder to detect differences in neuroplasticity. These findings offer initial insights into possible female versus male differences in white matter neuroplasticity during motor learning. This warrants investigations into specific patterns of white matter neuroplasticity for females versus males across the lifespan. Understanding biological sex-specific differences in white matter neuroplasticity may have significant implications for the interpretation of change associated with learning or rehabilitation.

## Introduction

Neuroplasticity is a lifelong process where the brain reorganizes neural networks based on aging, life experiences, and directed practice and learning^1–4^. Motor learning is the process by which new motor skills are obtained and refined^5,6^. While the concept of neuroplasticity and the fact that the brain has a lifelong capacity to change is well accepted, the underlying mechanisms involved in this process are not yet fully understood. Multiple imaging methods appear to be sensitive to the underlying processes activated during neuroplasticity^7^.

Magnetic resonance imaging (MRI) can be used to non-invasively characterize change in both structural and functional neuroanatomy^8–15^. MRI-derived diffusion tensor imaging (DTI) investigations of experience-dependent changes have provided in-vivo evidence for microstructural changes, specifically in white matter (WM)^1,9,10,12,13,16–19^. MRI-derived myelin water imaging (MWI) demonstrates histological accuracy as an in-vivo myelin measurement technique^20,21^, exhibiting sensitivity across diverse groups^22–24^. Moreover, it complements diffusion tensor imaging (DTI)^25^ as another MRI-derived modality to detect changes in myelin levels^10^.

The cortico-spinal tract (CST) is the primary descending pathway for voluntary motor control in the human central nervous system. Approximately 90% of the CST crosses the midline in the brainstem to provide control for contralateral portions of the body^26–29^. Reid et al. trained participants in a motor task with their left hands and examined MRI changes in the contralateral right hemisphere. The authors reported increases in fractional anisotropy (FA) along the right contralateral CST, measured with DTI^12^. Our group found similar results after training participants to perform a motor maze tracking task with both their left and right hands^8–10^. Participants showed significantly improved speed and accuracy with their non-dominant left hands, but not their right. We also noted structural and functional changes in the contralateral hemisphere, but not in the ipsilateral hemisphere^8–10^. Other recent MRI studies have shown contralateral neuroplasticity changes associated with motor learning (i.e., brain changes in the hemisphere contralateral to the trained limb)^8–12,30^. Yet no work to date has considered whether neuroplastic change associated with motor learning differs between biologically female and male individuals.

Biological sex differences may influence the mechanisms that underly neuroplastic change^31,32^. Both estrogen and testosterone affect neuroplasticity^33–36^. Catenaccio et al. completed a systematic review of neuroimaging literature and discovered physiological variation of brain macro- and microstructure in females caused by menstrual cycle phase, hormonal contraceptives, and menopause. Although generalizing findings across studies is difficult due to sample and methodology heterogeneity, the Catenaccio review concluded that ovarian hormones drive neuroplasticity^33^. Similarly, studies in mice have found that genetic background can have a large effect on learning, neuroplasticity, and behavior^37,38^. Additionally, genetic variation (specifically in regard to brain-derived neurotrophic factor) affects stroke rehabilitation^39,40^. Therefore, if subtle genetic differences can affect neuroplasticity, then they may also impact motor skill learning.

The use of non-invasive neuroimaging to investigate differences in female and male brains has increased over the past three decades. The majority of early studies researching human sex based differences in the brain have focused on the size of major brain structures, or the size of the brain as a whole^41–44^. Varied conclusions regarding sex differences in the corpus callosum (CC) have been drawn from DTI data^45^. FA values^46^ and relative anisotropy^47^ have been reported to be higher in male CCs. Other work showed higher FA in female CC splenium^48^ and different FA levels based on area of CC^49,50^, or no difference at all^51–53^. According to Toschi et al., WM degeneration associated with healthy aging in several regions, including the CC, begins earlier in males (> 10 years) than females, with males also showing higher FA values than females^45^. Interestingly, Schmithorst et al. used FA to show females (ages 5–18 years) displayed a trend of increasing organization with age (reflected in increasing FA values) only in the right hemisphere, while age matched males only showed this trend in the left hemisphere^48^. Yet it is not clear how to interpret higher FA in this context, as FA values in males did not correlate with improved cognitive function, while it did with females^54^.

The body of the CC has been associated with motor and visuomotor task activation^55–57^ and FA measures in this region positively correlate with performance during a motor learning session^58^. While past work has shown relationships between FA in the CST and CC, with measures of motor learning^8–12,30,59–63^ very little work has considered how biological sex impacts this relationship. This gap in our knowledge fundamentally limits our ability to tailor sex specific motor learning interventions. Even from a methodological perspective, it is important to better understand whether sex-differences represent a factor in WM neuroplasticity (and neuroplasticity in general).

**Figure 1:**
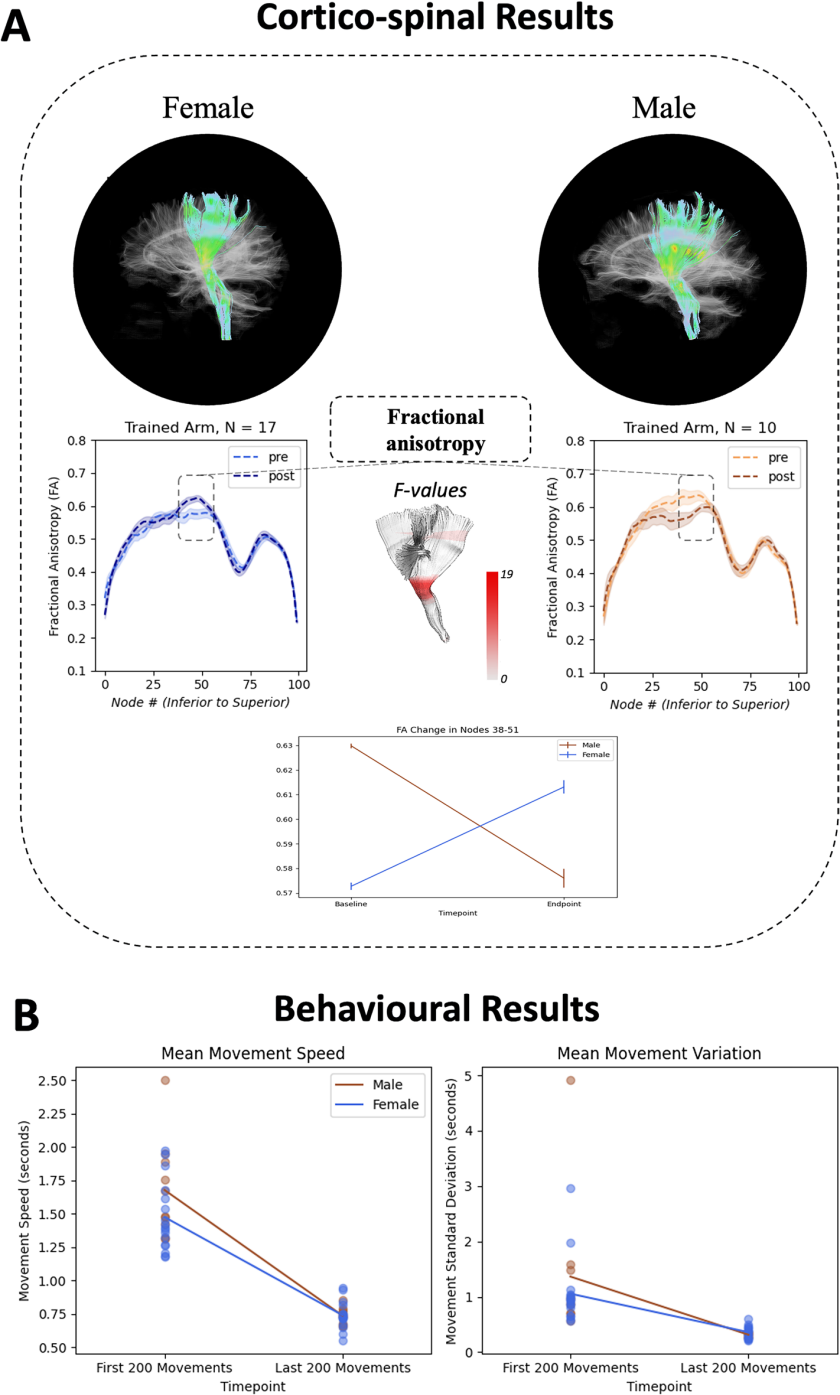
Cortico-spinal tract tractography results of representative female and male subjects and group mean tract profiles separated by sex (with standard error shading). Nodes used for repeated measures analysis of variance highlighted in dotted box and plotted. Additionally, color intensity mapping of *F*-values from cluster-based permutation method are portrayed on the model cortico-spinal tract at approximate node locations (**A**). Change in average speed and standard deviation of speed for all participant movements from the first 200 movements to last 200 movements (**B**).

Thus, the current study aimed to characterize differences in neuroplastic change in female and male CST and CC regions associated with learning a complex semi-immersive, visuo-motor task. Given the prior WM microstructural MRI evidence, we focused on FA and myelin water fraction (MWF) differences. We predicted that underlying FA and MWF differences between females and males would be significantly detectable in the CST and CC, and specifically for the CST these differences would be evident in the contralateral hemisphere following motor learning with the non-dominant hand (Study Summary - Fig. 1).

## Results

### Behaviour

**Figure 2:**
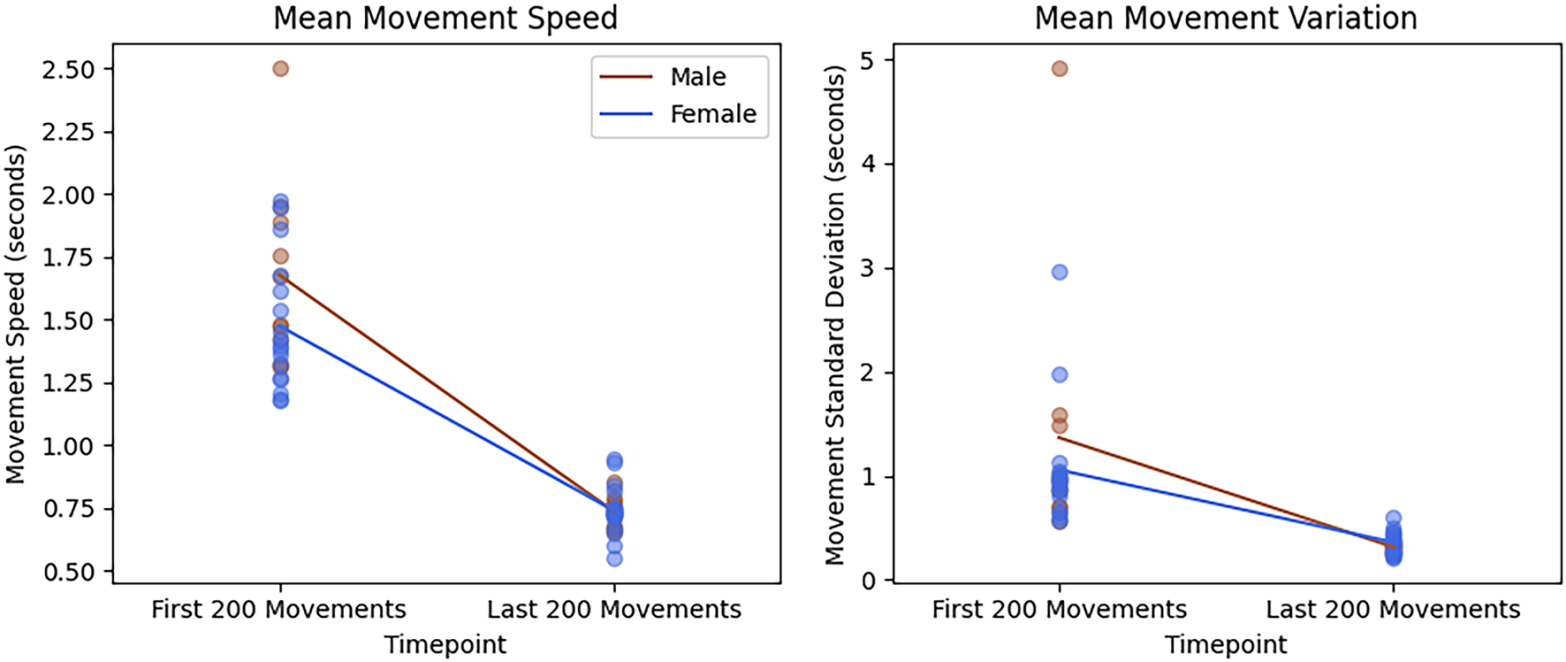
Change in mean movement speed and in mean movement speed variability in seconds from the first 200 movements to the last 200 movements. Results are colour coded by female (blue) and male (brown). Both dependent variables showed a significant main effect of time (*p* < 0.001).

Across 10,000 trained arm movements, participants showed significant decreases in mean movement speed and movement speed variability (decrease in movement speed standard deviation) from the first 200 movements to the last 200 movements (Fig. 2). At baseline, female and male mean movement speed and mean movement variation had equal variances [based on Levene’s: Speed: *F*(1,25) = 0.970,* p* = 0.33. Variance: *F*(1,25) = 0.945,* p* = 0.34]. Therefore, a two-tailed independent samples t-test determined baseline mean movement speed and baseline mean movement variation were not significantly different between sexes [speed: *t*(25) = 1.684, *p* = 0.105, *g* = 0.651; and variation: *t*(25) = 0.859, *p* = 0.399, *g* = 0.332]. Significant main effects of time were observed for movement speed [*F*(1,25) = 195.41, *p* < 0.001, η_p_^2^ = 0.887] and movement speed variability [*F*(1,25) = 24.838, *p* < 0.001, η_p_^2^ = 0.498]. However, the sex × time interaction was not significant for either dependent measure [Speed: *F*(1,25) = 2.952, *p* = 0.098, η_p_^2^ = 0.106 & Variability: *F*(1,25) = 1.027, *p* = 0.321, η_p_^2^ = 0.039]. Additionally, female and male ages had equal variances [based on Levene’s: *F*(1,25) = 0.069,* p* = 0.796]. A two-tailed independent samples t-test determined that female and male participant groups did not show a significant age difference [*t*(25) = 1.170, *p* = 0.253, *g* = 0.452].

### Neuroimaging

#### Cortico-spinal tract

**Figure 3:**
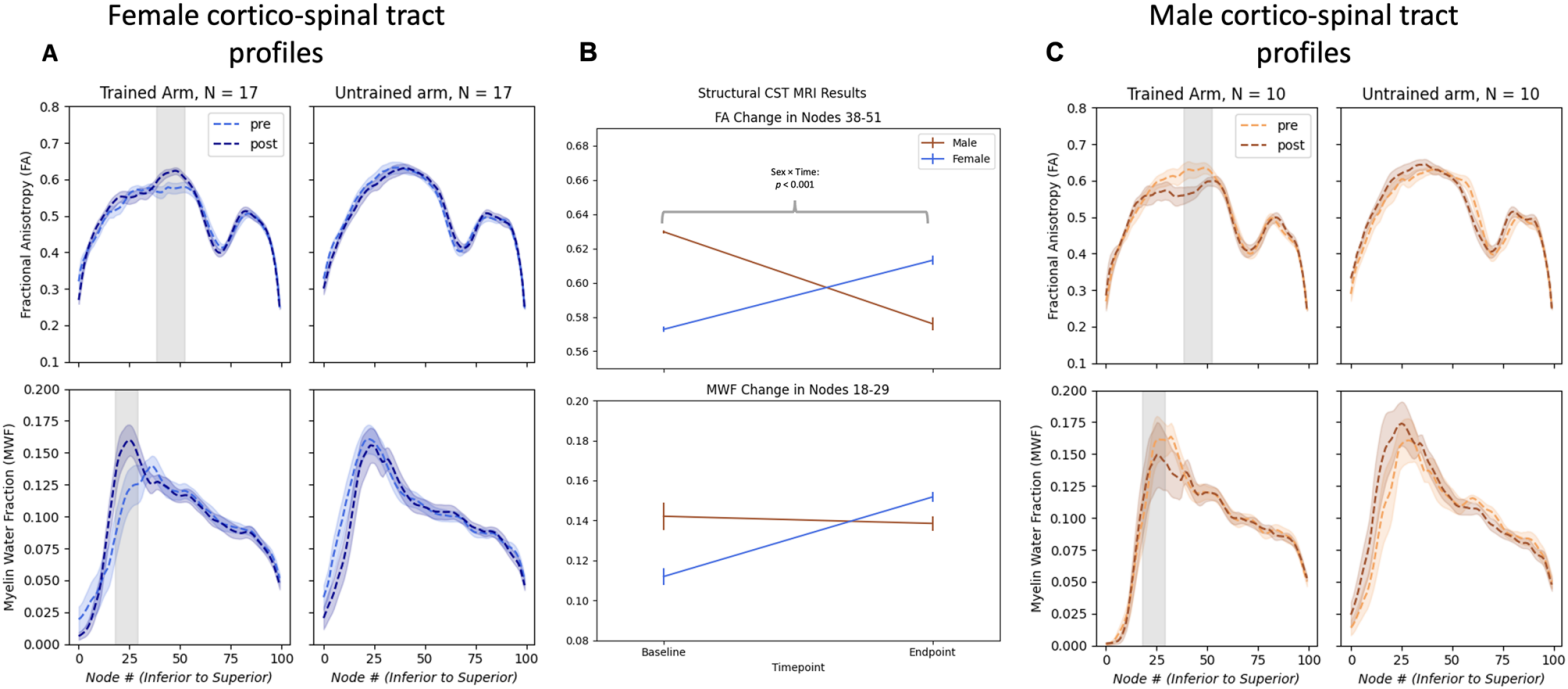
Cortico-spinal tract fractional anisotropy (FA) and myelin water fraction (MWF) profiles at baseline and endpoint for both the tract pertaining to the trained arm and untrained arm. Standard error shading is included. Grey shaded region covers the nodes that were then used in the mixed repeated measures analysis of variance (Female: **A**; Male: **C**). Mean FA and MWF changes in grey shaded nodes (FA: 38–51; MWF: 18–29) for females and males (**B**).

FA and MWF tract profiles of CST contralateral to the trained non-dominant arm showed sex-specific changes from pre- to post-behaviour training along the tract. Cluster-based permutation testing of the FA tract profile showed significant change in both female and male separated profiles in the middle portion of the CST tract (14 nodes^38–51^﻿, cluster-based approach *p* < 0.005) (Fig. 1). A mixed repeated measures analysis of variance (RM-ANOVA) did not show a significant main effect of time [*F*(1,25) = 0.348, *p* = 0.561, η_p_^2^ = 0.014] or sex [*F*(1,25) = 0.364, *p* = 0.552, η_p_^2^ = 0.014], but showed a significant sex × time interaction in this area for FA [*F*(1,25) = 17.109, *p* < 0.001, η_p_^2^ = 0.406] (Fig. 3). Additionally, post-hoc testing showed significant change in this cluster for both females and males (females: *p* = 0.007, males: *p* = 0.006, *Bonferroni corrected*). The MWF tract profile only displayed a non-significant cluster of 12 nodes in the inferior portion of the tract (nodes 18–29) for females. Examination of the results showed increased MWF variability, particularly in the male CST.

### Corpus callosum

**Figure 4:**
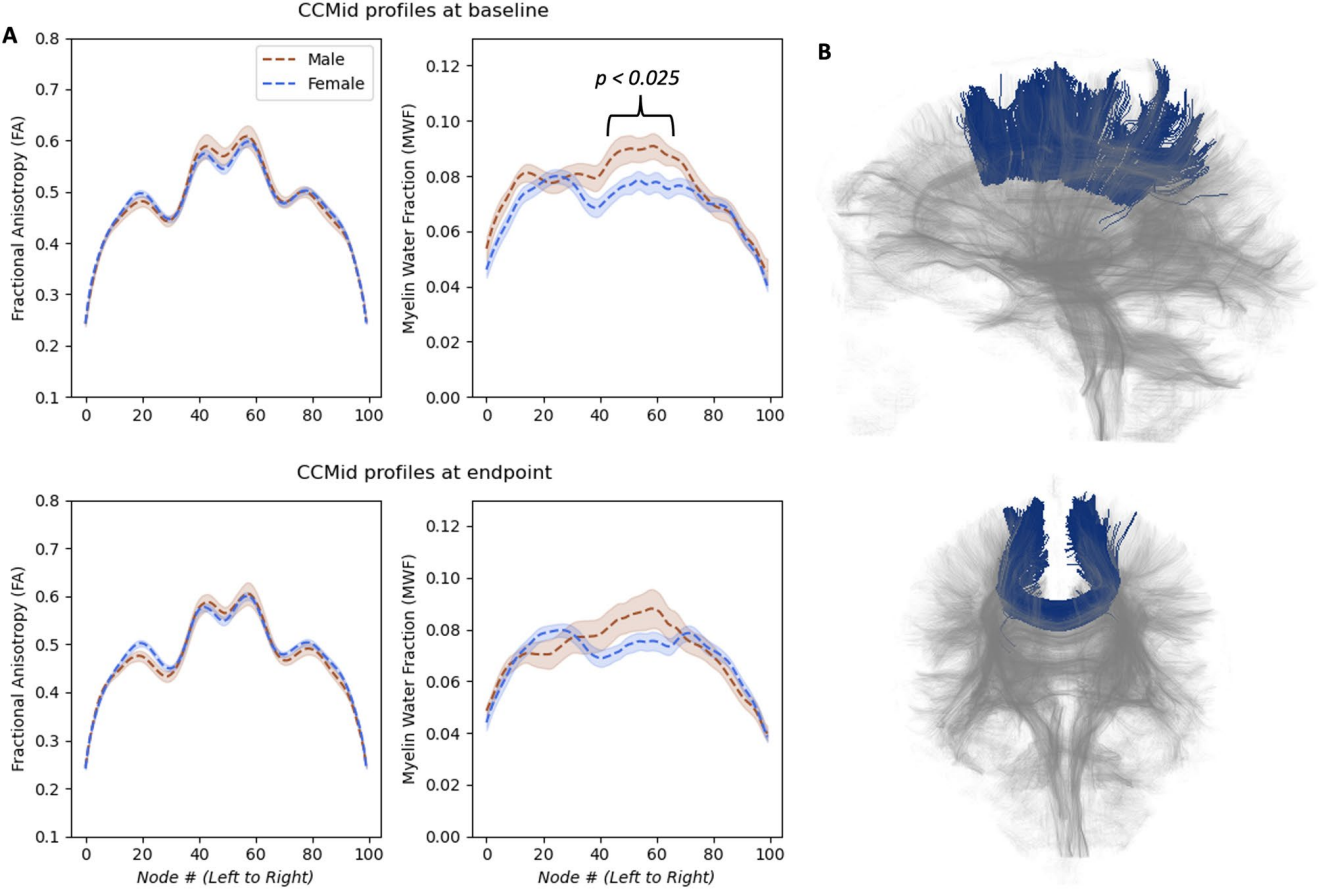
Baseline and endpoint myelin water fraction (MWF) and fractional anisotropy (FA) tract profiles of tracts traversing from left medial cortex to right medial cortex through the body of the corpus callosum (CC) for female and male brains. Shading represents standard error (**A**). A representative’s CCMid tractography result with faded whole brain tractography background (**B**).

While no main effect of time or sex × time interactions were observed in the CC for FA or MWF, there was a significant MWF difference at baseline between females and males. Males had greater MWF in baseline tracts through the body of the CC compared to females, specifically in the middle portion of the tract (26 nodes [nodes^42–67^,﻿ permutation test peak *p* < 0.025) and RM-ANOVA post-hoc testing showed a significant male greater than female difference at baseline in this area of the MWF tract [*t*(25) = 2.526, *p* < 0.025, *g* = 0.976, *Bonferroni corrected*] (Fig. 4).

## Discussion

The current study characterized changes in female and male brains associated with visuo-motor learning. We used a similar tract-based analysis to Kirby et al.^10^, which stemmed from original tract profile publications like Yeatman et al.^64^ and Dayan et al.^65^, to analyze data before and after complex visuo-motor training and directly evaluate the CST and the CC. The results of our analyses suggest structural differences between female and male patterns of neuroplastic change. Secondarily, the study demonstrated baseline CC tract (male greater than female MWF) differences that were not evident following motor learning.

To our knowledge, this is the first investigation to evaluate female and male neuroplastic change in WM using multimodal MRI. At minimum, the current study provided three noteworthy insights: (1) microstructural MRI measures of neuroplasticity in WM appear to be consistent with emerging evidence from the prior literature^8–12,30^, (2) the findings stressed the importance of disaggregating female and male data, and (3) sex related differences in WM were noted despite similar change in motor behaviour for females and males. Consistent with our past studies on WM neuroplasticity^9,10^, FA and MWF showed sensitivity to neuroplastic change in the CST contralateral to the trained limb, following behavioural change. Contrary to our previous findings, MWF did not reach significance in the current study. This may be due the older age in the current study, as MRI-based myelin levels have been previously associated with age^66,67^, or due to task related differences. As this complex task may have activated more networks, future studies can dig deeper into this potential more widespread change. Still, our findings highlight the importance of analyzing data by biological sex separately, as combining female and male data sets could result in a loss of significant WM findings, as shown by the absence of a significant time main effect in the RM-ANOVA. It is interesting to note that differences in structural WM were noted between females and males even though the pattern of behavioural change did not differ between the groups. The structural MRI evidence is consistent with fMRI studies of female versus male fMRI activation differences^68–73^. Gorbet et al.^72^ determined that performance between females and males in an increasingly complex visuo-motor task did not differ, but showed differences in fMRI activity between females and males. The current findings further underscore that differences may exist in one measurement modality and not the other. Future investigations are needed to characterize the underlying biological significance of these data.

The current study supports the importance of including data that are disaggregated by biological sex in analyses. Structurally, males have shown greater cortical surface areas in regions across the entire cortex than females. Importantly, males have greater variability in cortical surface area measures, as well as subcortical volume and cortical thickness, across the brain^74^. Furthermore, in studies with large numbers of participants, regional differences between females and males have shown that males have greater cortical surface area, while females show a greater cortical thickness for many regions across the cortex, including motor and somatosensory regions^74,75^. Less variability and increased thickness may lead to more focal neural processing. Functionally, this has been demonstrated by Andrushko et al.^73^ where greater functional volumes and larger inter-subject variability of fMRI activation was observed in males compared to females across 12 different voluntary motor tasks. Thus, a more focal and consistent neural signaling was noted in females during voluntary motor movements^73^. While not fully understood, these data hint at the possibility that different neural processes underpin brain activity in females versus males. It is possible, that in the current work more focused and efficient neural processing by females during motor learning led to neuroplasticity differences in WM. Speculatively, a more focal and efficient neural processing by females would not need an increased speed of signal transmission from a large cortical surface area, but instead would be focused on more direct WM structures for efficient signal transmission, such as the CST. This pattern of activity could be a potential explanation for the significant FA increase in the CST in females and not males; future work that contains both functional and structural data will have to test this hypothesis.

It is curious that at baseline MWF tract profiles of the CC body showed that males had more myelin as compared to females. At endpoint this difference no longer stayed significant. Others have reported similar data. Liu et al.^49^, Björnholm et al.^50^, and Shin et al.^46^ showed that males had higher FA as compared to females in multiple body portions of the CC. Altered myelination in the CC between females and males has previously been accredited to histological studies showing the total number and density of fibers to be decreased rather than increased in male CC regions^76^. As a result, Westerhausen et al. and Schmithorst et al. hypothesized that higher microstructural properties in male CC regions are due to fewer but thicker myelinated fibers^47,48^, further explaining higher MWF levels in males. It is known that thicker myelinated fibers increase the speed of signal transmission^77^. Thus, it is possible that greater myelination across the corpus callosum may be used to recruit a higher surface area of cortex to carry out voluntary motor tasks in males. However, at this time these ideas are speculative. As this field of research is still in its early stages, the underlying causes of microstructural and neuroplasticity differences between female and male brains are not yet fully understood.

Several issues may limit the generalizability of the data reported here. It should be noted that the female (n = 17) and male (n = 10) subsamples were not closely matched by number and they are relatively small. Accordingly, the current findings must be replicated with larger, matched sample sizes. Indeed, with higher sample sizes it may be possible to detect more subtle but significant changes in the corpus callosum between females and males. Furthermore, as sex hormones change across the lifespan, future work should include data from a large age range and incorporate reproductive factors that affect hormone exposure. An increased age range can also offer greater generalizability and potentially reveal different mechanisms of neuroplasticity across the lifespan between biological sex, as this biological sex and age connection has been explored in neuroplasticity and exercise efficacy^78^. The current findings were obtained from a specific task in targeted CST and CC WM structures. Complexity, task-specific goals, and baseline motor skills represent additional factors affecting WM neuroplasticity, as these have previously been shown to impact motor skill learning in general^79,80^. As such it is not clear whether the differences shown in the current data set will generalize to other motor learning tasks. Further, FA has also demonstrated low sensitivity to a single neurobiological process^81^ making it difficult to characterize the exact mechanism behind neuroplasticity changes, specifically the decrease in measures noted in males. Prior research investigating neuroplasticity has observed reductions in FA associated with learning. This has been interpreted as improved performance and learning-related FA or diffusivity decline according to previous studies^61^, while another study attributed decrease in μFA (a different form of FA) to synaptic pruning^82^. Future studies should integrate other non-MRI measures, such as magnetoencephalography, to further investigate female and male neural processing differences and the neuroplasticity that underlies this. Lastly, differences in patterns of change in females vs males should be investigated in patient populations, including stroke, to better understand neuroplastic mechanisms of recovery and to enhance the effectiveness of neurorehabilitation interventions.

## Conclusion

While not definitive, due to the limitations outlined above, our findings suggest that female and male differences in WM neuroplasticity may be detectable using MRI FA measures. MWF changes followed a similar trend but were insignificant. Importantly, we discovered differences in WM for females versus males despite both groups showing significant behavioural changes associated with motor learning. These findings suggest that there are distinct patterns of change that take place in female versus male brains to support motor learning. Critically, had the two groups been combined these WM different patterns would not have been detected. Despite increasing recognition in the field of neurosciences and health related research that sex should be considered in neuroscience study design and analysis^83,84^, most studies fail to do so^85,86^. Our behavioral and imaging results are in line with the demand of all funding agencies to include sex as a biological factor^86–88^ and provide further support for the importance of including sex-based analyses, as females and males may exhibit differences in neural mechanisms^45,89,90^. Our data suggest that future work should consider how differences in biological sex affect patterns of both structural and functional change associated with motor learning.

## Methods

### Experimental paradigm

The current study analyzed a healthy normative data set from a larger study that focused on stroke rehabilitation. Twenty-seven (N = 27) neurologically intact, older adult participants (mean age ± standard deviation: 64.2 ± 8.5 years, 2 left-hand dominant, 17 females, female mean age ± standard deviation: 62.8 ± 8.0 years, male mean age ± standard deviation: 66.7 ± 8.3 years) completed a 10-session (4 week) complex visuo-motor training task. Task specifics are provided in Kraeutner et al.^91^. Briefly, participants engaged in a semi-immersive virtual reality-based intercept and release task (TRack And Intercept Task; TRAIT) that was presented on a 46-inch monitor, viewed at 72 inches away (screen refresh rate 59 Hz). Movement time in seconds was recorded for all intercept and release movements during the visuo-motor training sessions using a Microsoft Kinect (model no. 1517, Kinect for Window; Microsoft, Redmond, WA) camera. The task was calibrated using a four-point grid to personalize the workspace. The task was set in “outer space”, where the spaceship, asteroid, and sun corresponded to the position of the hand, object, and target, respectively. The task was designed to create a semi-immersive engaging and motivating environment to promote a large dose (10,000 repetitions) of skilled movements^92^.

Participants trained with non-dominant arms by intercepting a virtual moving object and then accurately throwing the object at the target. Auditory feedback, visual feedback and knowledge of results were provided and used to maintain engagement. Each of the 10 sessions involved 5 blocks of the task, with each block containing 200 movements (100 object intercepts and 100 object releases). An 80% success rate for two consecutive blocks advanced the participant to the next level of difficulty. Increasing object speed, decreasing object size, and decreasing target size were used to manipulate task difficulty. Informed consent from each participant was obtained according to the Declaration of Helsinki. The Research Ethics Board at the University of British Columbia approved all study procedures.

### Behavioural statistical analyses

To investigate sex differences associated with motor learning, a mixed between (sex: female, male) and within (time: baseline and endpoint) RM-ANOVA was run for both mean movement time and mean movement time variability (standard deviation) for the first 200 and last 200 movements of the learning task (i.e., the first and last block of training). All RM-ANOVA tests were run through SPSS (IBM Corp., Armonk, NY)^93^. Additionally, the Levene test^94,95^ was performed on the two sex groups’ mean movement time and mean movement time variability to determine equality of variance. To ensure there were no differences at baseline between the two groups, two-tailed t-test was completed on mean movement time and mean movement time variability between the two biological sex groups. Lastly, the Levene test^94,95^ was performed on the two sex groups’ ages to determine equality of variance. To ensure there were no differences between the two groups, two-tailed t-test was completed on age between the two biological sex groups.

### Magnetic resonance imaging acquisition

MRI data were acquired at baseline and endpoint within 24 h before motor training and after completing four weeks of motor practice with a 3 Tesla Philips Achieva (Best, The Netherlands) MRI scanner using an eight-channel sensitivity encoding head coil and parallel imaging. MWI data were collected using a 32-echo gradient and spin echo (GRASE) MWI sequence T2 scan^96^. Parameters were as follows: TR = 1000 ms, TE = 10, 20, …, 310, 320 ms, scan duration = 836 s, 20 slices acquired at 5 mm slice thickness, 40 slices reconstructed at 2.5 mm slice thickness, resolution = 232 × 225, final dimensions: 240 × 240 × 40. DWI-based high angular resolution diffusion imaging (HARDI) data were acquired using a single-shot echo-planar imaging sequence with 60 diffusion directions, b-value of 700 s/mm^2^, and one b-value of 0 s/mm^2^ as the final volume of the scan. Acquisition parameters included: TR = 7088 ms, TE = 60 ms, scan duration = 447 s and voxel dimensions of 2 × 2 × 2.2 mm (70 slices), for tractography purposes voxels were reconstructed to isotropic size of 2 × 2 × 2 mm (77 slices) using FSL’s FLIRT^97,98^.

### Magnetic resonance imaging processing

MWF maps were created using DECAES^99^ with T2 relaxation distributions spaced from 10 to 2000 ms and a T2 distribution of 10 ms < T2 < 25 ms for MWF. Similar to Birkl et al.^100^ and Kirby et al.^10^, this range was determined by analyzing T2 distribution peaks in the data. MWF maps were then transformed to the individual’s native DWI (diffusion weighted imaging) space by registering the first echo of the GRASE MWI data to the non-diffusion weighted scan from the DWI data using FSL’s FLIRT^97,98^. The resulting transform was then applied to the individual’s MWF map.

DWI data were motion and eddy corrected using FSL^101^. DIPY^102^ workflows were used for tractography and tract profile processing steps^103^ that included FA map creation and whole brain deterministic tracking using EuDX tracking with constant solid angle peaks^104,105^. CSTs were extracted using RecoBundles workflow^106^ and a model atlas^107^. BUndle ANalytics (BUAN) similarity scores compared each participant’s left and right CST at endpoint to their baseline tract. Briefly, this determined difference in shape, length, and size of streamline tracts using a metric called bundle adjacency (0 = no similarity, 1 = perfect similarity)^103^. Any participants with a low BUAN score (< 0.7) for either their left or right CST comparison were removed to run statistical tests with and without these data. A similar pattern of results remained; therefore, the two participants’ results were kept. Similar to the CST analysis, the CC analysis used the CCMid tract from the Yeh atlas^107^ and Chandio et al.^103^. This tract looked at all tracts traversing from the left medial cortex to the right medial cortex through the CC body. There was no BUAN score < 0.7 for the CCMid analysis. DIPY was used to employ a similar technique to automated fiber quantification^64^ to create tract profiles of microstructural values at 100 equidistant nodes.

### Magnetic resonance imaging analysis

Changes in FA and MWF of females and males were evaluated across the right and left CST between baseline and endpoint scans using a cluster-based permutation method derived from^108,109^ to minimize the False Discovery Rate in mass univariate testing. Briefly, a mixed between (sex: female, male), and within (time: baseline and endpoint), RM-ANOVA was evaluated at each of the 100 nodes along the CST, specifically focused on sex × time interactions. Next, only resulting *F-*values below a threshold (*F*-value corresponding to *p* < 0.005) are used to generate clusters by identifying connected regions of significant data points. The result is a list of clusters and corresponding summations of *F-*values of each of these clusters, providing information about potentially significant regions in the data. Next, the data is shuffled randomly (sex and timepoint are shuffled between subjects) and rerun through this process. The maximum cluster sum of *F-*values along the 100 points is then stored for each iteration. This occurs for 5000 iterations to create the null distribution. The sum of *F*-values resulting from the clusters from the original RM-ANOVA are then compared to the array of maximum cluster summations of *F*-values, and only original clusters greater than the 97.5th percentile (two-tailed test) of the maximum cluster summations of *F*-values are considered to still be statistically significant. Results from *F-*max cluster-based testing were used as a searchlight for CST sections to run through a mixed between (sex: female, male), and within (number of nodes: FA = 14, MWF = 12; and time: baseline and endpoint), RM-ANOVA to evaluate both time and sex effects separately and as an interaction. Pairwise post-hoc comparisons done in the RM-ANOVA were corrected for multiple comparisons with Bonferroni correction. Non-parametric permutation testing was used to identify portions that were significantly different between female and male CCMid tracts at baseline. This portion was then run through a mixed between (sex: female, male), and within (number of nodes: MWF = 26; and time: baseline and endpoint), RM-ANOVA for consistency between tract analyses.

### Ethics statement

Informed consent from each participant was obtained according to the Declaration of Helsinki. The Research Ethics Board at the University of British Columbia approved all study procedures.

## Data Availability

The raw data used in the current study was collected with informed consent of the participants, who agreed to data use outlined by the research ethics boards of the University of British Columbia. Sharing of this data, supporting the conclusions of this article, will be made available by the authors, without undue reservation, to any qualified researcher. However, it is not currently available in any publicly accessible source. Any interest in receiving the data or post-processing code should be expressed to the corresponding author.
